# Heterogeneous Photocatalysis of Amoxicillin under Natural Conditions and High-Intensity Light: Fate, Transformation, and Mineralogical Impacts

**DOI:** 10.3390/environments9070077

**Published:** 2022-06-24

**Authors:** Nishanthi Ellepola, Gayan Rubasinghege

**Affiliations:** Department of Chemistry, New Mexico Institute of Mining and Technology, Socorro, NM 87801, USA;

**Keywords:** amoxicillin, environmental pharmaceuticals, heterogeneous photocatalysis, mineralogy, anatase, kaolinite

## Abstract

The β-Lactam antibiotic amoxicillin is among the most widely used antibiotics in human and veterinary medicine. Consequently, amoxicillin is abundant in natural waters and can undergo diverse abiotic reactions to form degradation compounds under environmental conditions. Yet, little is known about these decay pathways and mineralogical impacts on environmental amoxicillin degradation. The current study focuses on understanding the mineralogical influences of amoxicillin degradation under ecological conditions. We studied the role of anatase and kaolinite on amoxicillin degradation under irradiated and non-irradiated conditions. Anatase increases amoxicillin degradation by 4.5-fold in the presence of light compared to just being exposed to sunlight. Interestingly, anatase also showed a higher degradation rate under dark than light controls. Conversely, kaolinite diminishes the amoxicillin degradation under irradiation. The formation of degradation compounds was mineralogy-controlled, while no mineralization was observed. Further, we irradiated amoxicillin with a high-intensity light to evaluate its removal from wastewater. The formation of varying amoxicillin degradation products with high-intensity light will limit its removal from wastewater. Our study emphasizes that the mineralogical impact on amoxicillin degradation is diverse, and the role of anatase is significant. Consequently, the increased addition of manufactured titanium nanoparticles to the environment can further enhance these effects.

## 1. Introduction

Pharmaceuticals in the environment are a recurring problem that threatens human health and ecosystem balance due to their widespread availability. Pharmaceuticals are designed for specific therapeutic functions and may cause unexpected physiological effects on non-target species [1]. The potential consequences of these compounds in the environment have to be considered seriously, and growing attention has been directed to pharmaceuticals in ecological systems. The primary pathway for pharmaceuticals to penetrate the environment is direct disposal by hospitals, industrial, and domestic sewage. Additional sources of drug pollution are represented by metabolites, mainly via human and veterinary excretion [2–5]. Given that only about 25% of antibiotics are effectively used in aquaculture, the animal body poorly absorbs most antibiotics, and 30–90% of antibiotics are excreted via urine and feces into the environment. Consequently, antibiotics are frequently detected in surface water, groundwater, soils, and sediments [1,6,7].

Amoxicillin, a semi-synthetic broad-spectrum β-lactam antibiotic, is one of the most widely used antibiotics in the world. This drug is widely used in veterinary practices and can reach the environment primarily via aquaculture, livestock breeding, or human and veterinary excretion. It has been used extensively for disease control and livestock feed for several decades due to its excellent therapeutic value [1,4,7,8]. As a result, livestock agriculture is considered a significant source of amoxicillin pollution. Human excretion is recognized as another major entry point. Previous reports indicate that amoxicillin is poorly metabolized in the human body, and consequently, 40–80% of unmetabolized amoxicillin is excreted after oral application [2,6,9].

The continuous input of amoxicillin and poor metabolism in the human body should result in a relatively high concentration of amoxicillin in wastewater and surface water. Conversely, the reported amoxicillin concentrations in wastewater are at low levels [10,11]. Previous reports indicate that the environmental concentrations of amoxicillin are typically low in raw sewage, which can vary from several ng L^−1^ to μg L^−1^ [1,9,12,13], except some rare cases showed mg L^−1^ levels [12]. The infrequent detection of amoxicillin may be explained by a short retention time of amoxicillin in the environment due to the highly reactive β-lactam ring, which can be broken under environmental conditions and transformed into different products [4,6,9–11,14]. On the other hand, these compounds are tremendously challenging to trace due to the too low sensitivity of the analytical methods and lack of standards [4,10,15].

The fate of amoxicillin in the environment can be influenced by various factors, such as the physicochemical properties of the amoxicillin and the characteristics of the receiving environment. It can thus undergo degradation via various biotic, involves microorganisms mediated degradation of the organic compound, and abiotic pathways. Photolysis, hydrolysis, and chemical oxidation in surface waters are the most critical abiotic processes to convert amoxicillin into various secondary compounds [4]. Certain degradation products of amoxicillin have been found in the natural environment. A major degradation product, amoxicillin diketopiperazine, was confirmed in Israel wastewater effluents [10] and Spain river water [15] samples. These degradation compounds may exhibit more persistent and more hazardous than amoxicillin. For example, amoxicilloic acid, one of the primary degradation products of amoxicillin, retains allergenic properties that can cause adverse reactions in sensitive individuals [9].

A handful of studies have reported their attempts to identify amoxicillin degradation compounds under various simulated environmental conditions [14,16]. These studies have no or limited focus on the mineralogical impacts of amoxicillin degradation. The environmental minerals contain catalytic surfaces, which can result in different degradation products and decay rates of amoxicillin in the environment in combination with sunlight and water. Therefore, the current work’s most considerable interest is understanding potential mineralogical impacts on environmental amoxicillin degradation. Here, we selected two common environmental minerals, anatase and kaolinite. Anatase (TiO_2_) is a semiconductor mineral, present in suspended soil particles, with high photocatalytic activity [17,18]. On the other hand, kaolinite was selected as a proxy for the soil clay minerals [7,19–22].

Continuous exposure to low, subtoxic concentrations of amoxicillin can cause unexpected consequences and unintended effects on non-target species, such as the development of antibiotic-resistant microbial strains [8,13] and some negative impacts on the growth of several algal species in the environment [23]. Chronic exposure to antibiotics or other toxicants, along with antibiotics, may exert pressure on the evolution of antibiotic-resistant bacteria, which has become a significant environmental concern [3,4,7]. Furthermore, the bioaccumulation of small quantities of antibiotics may cause severe problems for humans and animals in the long term [4]. Therefore, amoxicillin can be recognized as a significant environmental contaminant. Due to its high usage in food-producing animals, amoxicillin residues can be present in food, leading to long-term health problems in humans and the spread of drug-resistant microorganisms. To ensure the safety of food for consumers, the European Union (EU) Commission has laid down maximum residue limits of 50 ppb of amoxicillin in animal tissues and four ppb in milk [24]. Additionally, degradation products of amoxicillin can enhance different interactions among diverse species within the ecosystems. These interactions can have more adverse effects on humans than parent amoxicillin. Yet, the amoxicillin degradation products’ occurrence, persistence, and toxicological effects are poorly understood. Although our current work does not focus on reporting such toxicological impacts, the results of this study can be exploited to make confident predictions of those issues in future studies.

Several chemical and biological methods for antibiotic removal from wastewater sludge have been proposed. These approaches include chemical processes such as ozonation, solid-phase separation, flocculation, coagulation, ultrasonic degradation, photodegradation, electrochemical degradation, and advanced oxidation processes [25,26]. Antibiotic degradation via biological processes has also been proposed [25]. These technologies suffer from various flaws as they are technically sophisticated or economically unfavorable. Moreover, these processes can result in antibiotic degradation products, potentially more toxic than the parent compound [14]. Therefore, the current work further focuses on mineralogy-controlled or mineralogy-dependent amoxicillin degradation with a high-intensity light to access such methods to remove amoxicillin from wastewater. Overall, the current study reports the photodegradation of environmental amoxicillin and the formation of degradation products, which are highly mineralogy-dependent.

## 2. Materials and Methods

### 2.1. Materials

Amoxicillin (98% purity) was purchased from the TCI chemicals and used without further purification. Amoxicillin solution of 1 mM was prepared using double DI Milli-Q water (Res > 18.2 MΩ, Millipore Advance A10, MilliporeSigma, Burlington, VT, USA). Anatase and kaolinite KGa-1b were purchased from the Source Clay Repository in Washington County, Georgia, USA, as proxy soil minerals.

### 2.2. Particle Characterization

The shape and size of mineral particles were determined from single particle analysis with a scanning electron microscope (SEM) and a transmission electron microscope (TEM). The detailed experimental protocols are provided in our previous work [27–29]. The size distribution was determined by analyzing *~*800 particles using the software package ImageJ. Surface areas of mineral samples were measured in a seven-point N_2_-Brunauer-Emmet-Teller (BET) isotherm using a Quantachrome Autosorb-1 surface area analyzer. Samples were outgassed overnight (*~*24 h) at a temperature of 105 °C prior to the BET analysis. X-ray diffraction (XRD) of mineral proxies was measured in an X’Pert Pro Diffractometer (Malvern Panalytical, Westborough, MA, USA) equipped with a Cu source. Sample spectra were compared with reference XRD using ICDD database provided by International Centre for Diffraction Data. Attenuated total reflectance-Fourier transform infrared (ATR)-FTIR spectra of samples were obtained using a Nicolet IS50 spectropho-tometer (Thermo Fisher Scientific, Waltham, MX, USA), equipped with a liquid-nitrogen cooled mercury–cadmium-telluride (MCT) detector and Ge ATR element.

### 2.3. Degradation Studies

#### 2.3.1. Batch Reactor Studies

The experiments were conducted under a nitrogen environment in custom-built glass reactors with a suspension capacity of 100 mL and a removable air-tight cap with a quartz window on the top. The quartz window (12.5 cm^2^) permitted light entry during the solar experiments. During light experiments, the solution surface received a solar flux of ~105 mW/cm^2^ and remained constant throughout reaction time. During the experiments, 1 mM amoxicillin solutions were used. Due to the difficulty in detecting transformed products in the final mixture, a high initial concentration of amoxicillin was used. On the other hand, these compounds are tremendously challenging to trace due to the low sensitivity of the analytical methods and lack of standards. Further, the added amoxicillin can be continuously transformed into other products leading to lower reported values. Therefore, concentrations of amoxicillin in the natural environment can be at higher levels than we used in the current study. These experiments were conducted in the presence and absence of a solar simulator (150 W xenon lamp, New Port Corporation, Irvine, USA) with Air Mass 1.5G and Air Mass 0 filters. The temperature was kept constant throughout the experiment using a water jacket. The particle loading was maintained at 0.1 g L^−1^ of anatase and kaolinite in solutions of 1 mM amoxicillin. The pH of the amoxicillin solution was allowed to vary freely during the experiment and was measured every 24 h (Thermo Orion, model 9272, Thermo Fisher Scientific, Beverly, MA, USA, equipped with an Orion Ross combination electrode). Over 14 days, samples were removed at 24 h intervals from the reactor using a disposable syringe connected to 12 cm of Teflon tubing. Aliquots (1.5 mL) were collected into HPLC vials after passing through a 0.2 μm PTFE filter (Cole-Parmer, Vernon Hills, USA). The filtered extracts were analyzed by reverse-phase HPLC (Agilent HPLC 1200 series, Eclipse Plus C18 Column, UV detector). The HPLC analyses were performed in isocratic mode with mobile phase 15% acetonitrile and 85% sodium phosphate buffer at 1 mL min^−1^ flow rate. The wavelength of the UV detector was set to 220 nm. The HPLC validation data for linearity is provided in Figure S5 in Supporting Information.

#### 2.3.2. Liquid Chromatography-Mass Spectroscopy (LC-MS)

Filtered samples collected at the end of the batch reactor experiments were analyzed using LC-MS to identify the amoxicillin degradation products in the reaction mixture. All LC-MS experiments were performed at the Montana State University Proteomics, Metabolomics, and Mass Spectrometry Facility. An Agilent 1290 LC was coupled to an Agilent 6538 quadrupole time-of-flight mass spectrometer. Liquid chromatography was performed on Waters Acquity C_18_ HSST3 (1.8 μm particle size, 2.1 × 100 mm) column maintained at 50 °C. The mobile phases consisted of water (0.1% formic acid) as solvent A and acetonitrile (0.1% formic acid) as solvent B. The initial mobile phase composition of 95% A was maintained for 1 min, followed by a linear gradient to 95% B over 4 min, held at 95% B for 4.5 min, and returning to 95% A for 0.5 min. A flow rate of 0.6 mL min^−1^ was used. The LC eluent was coupled to an electrospray ionization source operating in the positive-ion mode. The mass spectrometer was operated at 1 Hz over a mass range of 50–1000 *m*/*z*.

### 2.4. Statistical Analysis

All dark and light experiments were conducted at least in triplicate with average measurements reported. The kinetic of amoxicillin degradation is modeled as the pseudo-first-order using the equation ln C/C_0_ = −kt.

## 3. Results and Discussion

### 3.1. Particle Characterization

Surface and bulk characterization of the mineral particles provides essential information on their particle size, shapes, crystal phases, and purity. As reported in our previous work [27,28], anatase (TiO_2_) has an average particle size of 25 (±3) nm, determined from SEM and TEM images. That of kaolinite was 46 (±5) μm. The specific surface areas of anatase and kaolinite were 59 (±2) m^2^ g^–1^ and 10.1 (±0.1) m^2^ g^–1^, respectively. SEM image showed that the clays are highly irregular in shape. XRD patterns of mineral samples are in good agreement with their respective reference XRD patterns, confirming no phase change or contamination. ATR-FTIR spectrum of kaolinite showed the absorption bands at 3750–3550 cm^−1^, confirming the presence of surface hydroxyl groups [29].

### 3.2. Photo-Degradation Studies

The photodegradation of amoxicillin was measured in the presence of anatase and kaolinite under dark and irradiated conditions. A solar simulator was used as the light source. Samples were irradiated with terrestrial solar spectrum using AM 1.5G filter and high-intensity light using AM 0 filter. The solar spectrum corresponding to each filter is provided in Figure S6 in Supporting Information. The kinetics of amoxicillin degradation is modeled as the pseudo-first-order using the equation ln C/C_0_ = −kt to calculate initial decay rates for the first 150 h [30–32]. All the observed rate constants, half-life, and R_2_ values are given in Table 1. The kinetic data with fitted lines are provided in Figure S2 in Supporting Information. It is essential to mention that heterogeneous processes can be more complex than a simple first-order reaction, including the formation of adsorption complexes, equilibrium reactions among adsorbed and “free” chemical species, desorption, and many more. The observed minor deviations from the linearity for some experimental conditions in Figure S2 could be due to these processes, which are unaccounted for in the pseudo-first-order model. The reactions among adsorbed complexes are challenging to evaluate, and we suggest them for future studies.

### 3.3. Amoxicillin Degradation with Terrestrial Solar Spectrum (AM 1.5G Filter)

**Figure 1.**
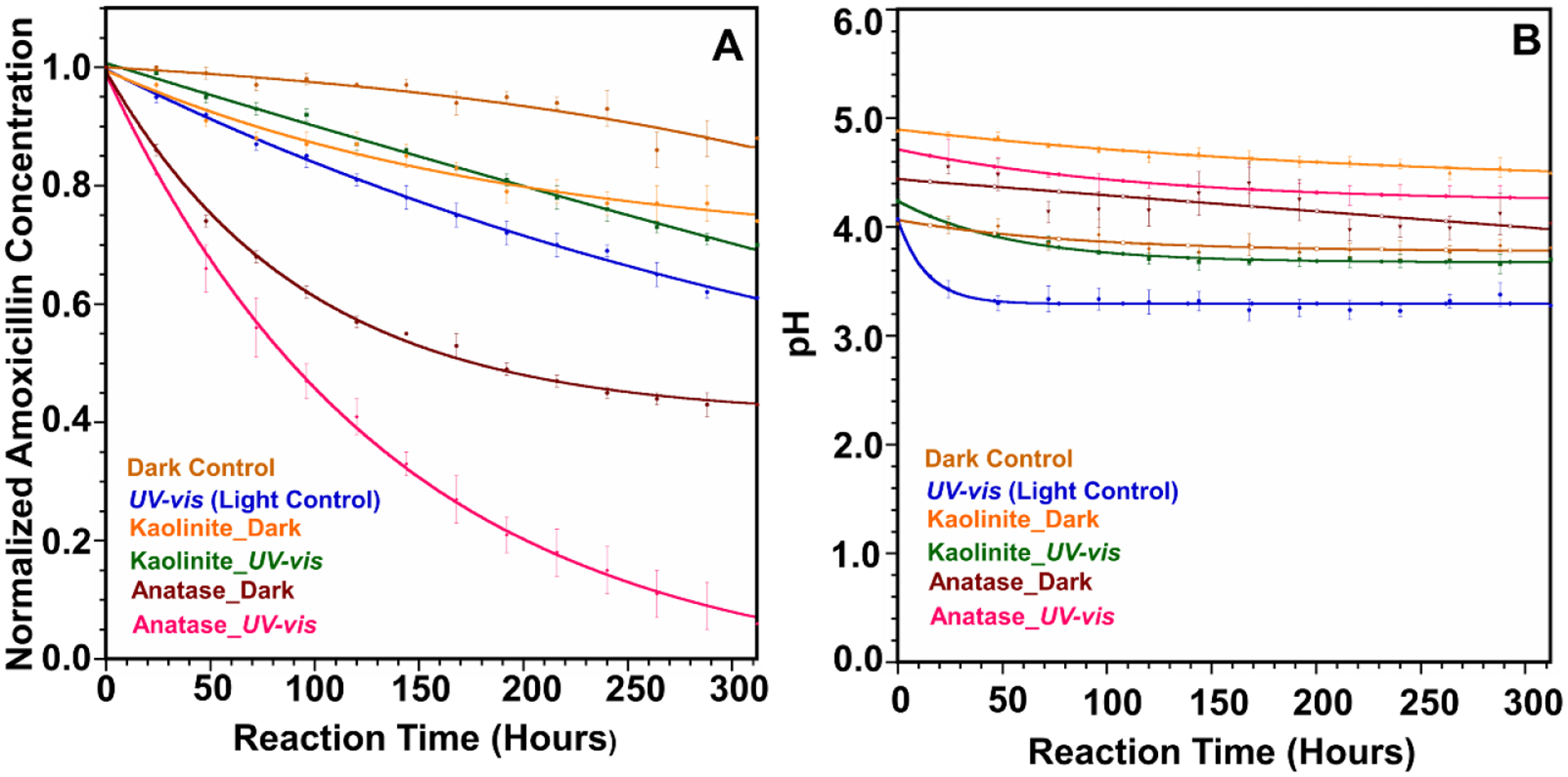
A comparison of amoxicillin decay under various experimental conditions. (**A**) Decay curves of amoxicillin with anatase and kaolinite under light and dark conditions. (**B**) pH variation of the amoxicillin with different experimental conditions.

The photodegradation of amoxicillin under terrestrial solar light is shown in Figure 1A. The contribution of the direct photolysis on the amoxicillin degradation in the experimental conditions adopted was relatively low. In the “Light Control,” which does not contain any mineral, only 30% of amoxicillin was degraded after 14 days of irradiation (Figure 1A). The rate constant and half-life for the Light Control were 0.00174 h^−1^ (R2 = 0.994) and 398 h, respectively. The observed low photodegradation of amoxicillin can be attributed to little or no overlapping between the amoxicillin light absorption profile (<280 nm) and the effective near UV/vis irradiation reaching the aqueous solution (>300 nm). Previous reports claim that amoxicillin is relatively stable and shows resistance to photolytic transformation in the presence of wavelengths above 300 nm [23,32,33]. Besides direct photolysis, amoxicillin can degrade via hydrolysis. This pathway proceeds via an attack by H_2_O, acting as a nucleophile to the β-lactam ring, followed by ring-opening [32,34]. Thus, a mechanism combining direct and indirect photodegradation of amoxicillin is proposed in the current study. For direct photodegradation, amoxicillin absorbs irradiation to reach a singlet-excited state. It then undergoes intersystem crossing to a triplet excited state, which leads to the transformation of amoxicillin molecules to degradation products, as shown in Equations (1) and (2). For indirect photodegradation, amoxicillin molecules are supposed to react with hydroxyl radicals according to Equations (3)–(5).

Direct photodegradation:

(1) $$\text{AMO}+\text{h}v\to^{1}{\text{AMO}}^{*}\to^{3}\text{AMO}$$

(2) $${\;}^{3}{\text{AMO}}^{*}\to \text{ Products }$$

Indirect photodegradation:

(3) $$\text{AMO}+\text{h}v\to^{1}{\text{AMO}}^{*}\to^{3}\text{AMO}*\;$$

(4)  3AMO*+H2O→AMO+O•H

(5) AMO+O•H→ Products 

where AMO is the ground state of pollutant, ^1^ AMO * is the singlet-excited state of AMO, ^3^ AMO * is the triplet excited state of AMO [35].

It is also important to mention that the Dark Control showed 10% amoxicillin degradation (Figure 1A), which could be attributed to the hydrolysis reaction taken place by the nucleophilic attack on the beta-lactam ring from water molecules. Interestingly, after introducing the anatase into the reaction mixture with light, the degradation rate of amoxicillin increased 4.4-fold compared to the Light Control, showing the degradation rate 0.00773 h^−1^ (R_2_ = 0.992) with a half-life of 89.6 h. Rapid decay of more than 70% of the initial concentration of amoxicillin (Figure 1A) was observed during the first 150 h of irradiation with anatase. The increased degradation rate reveals the “catalytic effect” of anatase surface during the amoxicillin degradation.

Anatase is a semiconductor having a bandgap of 3.2 eV. The bandgap is the energy difference between the valence band (VB) and the conduction band (CB). It relates to the photon energy required to excite the electron from the VB to the CB to create an electron–hole pair (e^−^ and h^+^) [12,32,33,36,37]. During photocatalytic reactions, electrons and holes on the surface of a semiconductor participate in redox reactions that produce reactive species (ROS) such as hydroxyl radicals (^•^OH) and superoxide anion radicals (^•^O_2_^−^) [32,34,36,37]. ROS reacts readily with surface adsorbed organic molecules, either by electron or hydrogen atom abstraction forming organic radical cations or by adding reactions to unsaturated bonds. Moreover, photogenerated holes can directly decompose the organic compounds [32,33,38]. The primary photocatalytic and redox reaction mechanisms are summarized in Equations (6)–(16) [32,36,39].


(6) $${\text{ Photoexcitation: TiO}}_{2}+\text{h}v\to {\text{TiO}}_{2}\left({\text{e}}^{-}+{\text{h}}^{+}\right)$$

(7)  Formation of hydroxyls with water: h++H2O→H++O•H

(8)  Formation of hydroxyls with holes: h++OH−→O•H

(9)  Photodegradation by O•H : organics +O•H→ Degradation Products 

(10) $$\text{ Direct photo holes degradation: organics }+{\text{h}}^{+}\to \text{ Degradation Products }$$

(11) Photo-excited electron scavenging: e−+O2→O•2−

(12)  Protonation of superoxide: H++O•2−→H•O2

(13)  Co scavenging of e−:H•O2+e−→HO2−

(14) $${\text{ Formation of H}}_{2}{\text{O}}_{2}:{\text{H}}^{+}+{{\text{HO}}_{2}}^{-}\to {\text{H}}_{2}{\text{O}}_{2}$$

(15) H2O2 breakdown: H2O2+e−→O•H+OH−

(16) $$\text{ Electron-hole recombination}{{\text{ e}}^{-}}_{\left(\text{TR}\right)}+{{\text{h}}^{+}}_{\left(\text{VB}\right)}\to {{\text{e}}^{-}}_{\left(\text{CB}\right)}+\text{ heat }$$

The current study was carried out under reduced conditions, and thus it is hard to claim the formation of superoxide radicals. Conversely, our experiments cannot expect Equations (11)–(15) in the above photocatalytic oxidation and reduction mechanism. The photodegradation of amoxicillin in the presence of TiO_2_ can be thus attributed to the reactive species, i.e., hydroxyl radicals and holes produced [32].

The inherent limitations of the broad bandgap for TiO_2_ (3.2 eV) will facilitate the electron–hole recombination according to Equation (16), which yields the low photo quantum efficiency, typically limiting its catalytic activity under visible light irradiation. In the absence of an electron scavenger, the photoexcited electron recombines with the valence band hole in nanoseconds [12,32,37]. Electron scavengers are vital in the photocatalysis of TiO_2_ to retard recombination. Often, oxygen acts as an electron scavenger, prevents the recombination of the electron–hole pair, and allows superoxide formation (O_2_^−^). Superoxide radicals can be protonated to form hydroperoxyl radicals (^*•*^HO_2_) that can react further to form H_2_O_2_ [32,36]. Interestingly, irradiation of amoxicillin with anatase showed a rapid decay even in the reduced environment. The pH of the reaction is considered an essential factor since it influences the surface charge properties of both anatase and amoxicillin.

Amoxicillin molecule possesses amphoteric properties (Figure S1) due to three main functional groups; COOH (pK_a1_ = 2.7), NH_2_ (pK_a2_ = 7.4), and OH (pK_a3_ = 9.6) [31,32,37,40,41]. Similarly, pH affects the surface charge of the catalyst, making it one of the most significant operating parameters in heterogeneous photocatalysis. The net surface charge of the catalyst can be positive or negative due to the amphoteric behavior of the metal oxide catalyst. The pH of the point of zero charge (pH_PZC_), the pH at which the surface charge of the catalyst is zero, helps define the effect of pH on the surface charge of the catalyst. Several studies have reported that the pH_PZC_ of TiO_2_ ranges between pH 6 and 8 [36]. For acidic conditions (pH < pH_PZC_), the surface charge of anatase is positive and increases the attraction for negatively charged contaminants. The anatase surface charge is negative for basic conditions (pH > pH_PZC_), attracting positively charged pollutants. Equations (17) and (18) show the protonation and deprotonation of TiO_2_ with pH.


(17) $$\text{pH}<{\text{pH}}_{\text{PZC}}:\text{TiOH}+{\text{H}}^{+}\to {{\text{TiOH}}_{2}}^{+}$$

(18) $$\text{pH}>{\text{pH}}_{\text{PZC}}:\text{TiOH}+{\text{OH}}^{-}\to {\text{TiO}}^{-}+{\text{H}}_{2}\text{O}$$

Figure 1B shows that the pH of the reaction mixture of amoxicillin in the presence of anatase varies from 4.7 to 4.2. The acidic environment is favorable for forming a positive charge at the surface. Meanwhile, amoxicillin owns both positive and negative charges in its structure, allowing more amoxicillin molecules to be adsorbed onto the photocatalyst surface. As a result, photogenerated holes will have less chance to undergo recombination. A large amount of amoxicillin adsorbed onto the anatase surface should be effectively degraded by ^*•*^OH radicals and photogenerated holes (h^+^). Previous reports claim that photogenerated holes (h^+^) are the dominant oxidizing species at low pH, where hydroxyl radicals play the dominant role in pollutant oxidation while under neutral or alkaline solutions [30,36,39,42]. Thus, photogenerated holes may play a significant role in the degradation of amoxicillin on the anatase surface. Besides, it is well known that TiO_2_ particles tend to form agglomerates when dispersed in aqueous media. Such agglomeration strongly depends on parameters such as ionic strength and the pH of the suspension. It has been reported that agglomeration of TiO_2_ particles in water decreases at acidic conditions compared to neutral or alkaline conditions, thus increasing the effective surface area of the catalyst [31,37].

Another possible pathway to retard the electron–hole recombination in an acidic medium is capturing electrons by hydrogen ions according to Equation (19) [27]. It further helps to prevent electron–hole recombination. These factors can collectively enhance the catalytic activity of anatase for amoxicillin degradation.


(19) $$2{\text{H}}^{+}+2{\text{e}}^{-}\to {\text{H}}_{2}$$

The overall process of heterogeneous photocatalysis can be decomposed into five steps. First, amoxicillin moves from the fluid phase to the interface region of the catalyst by diffusion. Second, amoxicillin is adsorbed onto the surface of the photocatalyst. Third, a chemical reaction occurs with oxidizing and/or reducing species in the vicinity of the surface. Fourth, by-products desorb. Finally, products are removed from the interface region into the bulk fluid [36].

It is worth highlighting that the catalytic effect of anatase for amoxicillin degradation was also significant under dark conditions. Figure 1A shows that adding anatase to the reaction medium increased the amoxicillin degradation rate by 19-fold compared to its Dark Control (no surface). The decay constant for Anatase_Dark was 0.00469 h^−1^ (R^2^ = 0.987), while the half-life was 148 h. Interestingly, the Anatase_Dark reaction showed a 2.2-fold increase in degradation rate compared to the Light Control. Figure 1B shows that the pH of the Anatase_Dark reaction changed from 4.4 to 4.0, during which the anatase surface was positively charged. Amoxicillin owns both positive and negative charges in its structure at this pH. This will lead more amoxicillin molecules to adsorb to the anatase surface. Further, hydroxyl radicals can rapidly react with the aromatic ring in the side chain of amoxicillin [42]. Hence, the observed rapid degradation of amoxicillin could be attributed to the catalytic effect of surface hydroxyl groups of anatase.

Conversely, the amoxicillin degradation was lower in kaolinite (band gap = 4.99 eV) [43] presence under irradiated conditions than the Light Control. The observed rate constant was 0.00105 h^−1^ (R^2^ = 0.997) with a half-life of 660 h. The photocatalytic effect of the kaolinite for amoxicillin degradation is insignificant due to limited light penetration in the presence of clay particles. However, the observed 20% amoxicillin loss over the 14 days could be attributed to the hydrolysis of amoxicillin via hydroxyl groups on the kaolinite surface. The pH of the point of zero charge (pH_PZC_) of kaolinite lies between 6 and 6.5 [44]. Figure 1B shows that pH of the kaolinite reaction under light varies from 4.5 to 3.8, and the kaolinite surface will be positively charged. Amoxicillin owns both negative and positive charges in its structure at this pH. Hence, amoxicillin can be adsorbed to the positively charged kaolinite surface via an ionized carboxylate group. This is further confirmed by the 20% amoxicillin loss in the presence of kaolinite under dark conditions (Figure 1A). Moreover, we observed a 5-fold increase in amoxicillin degradation compared to the Dark Control, which reveals the catalytic effect of the kaolinite surface to increase the hydrolysis of the β-lactam ring.

### 3.4. Amoxicillin Degradation Mechanism under Terrestrial Solar Radiation

**Figure 2.**
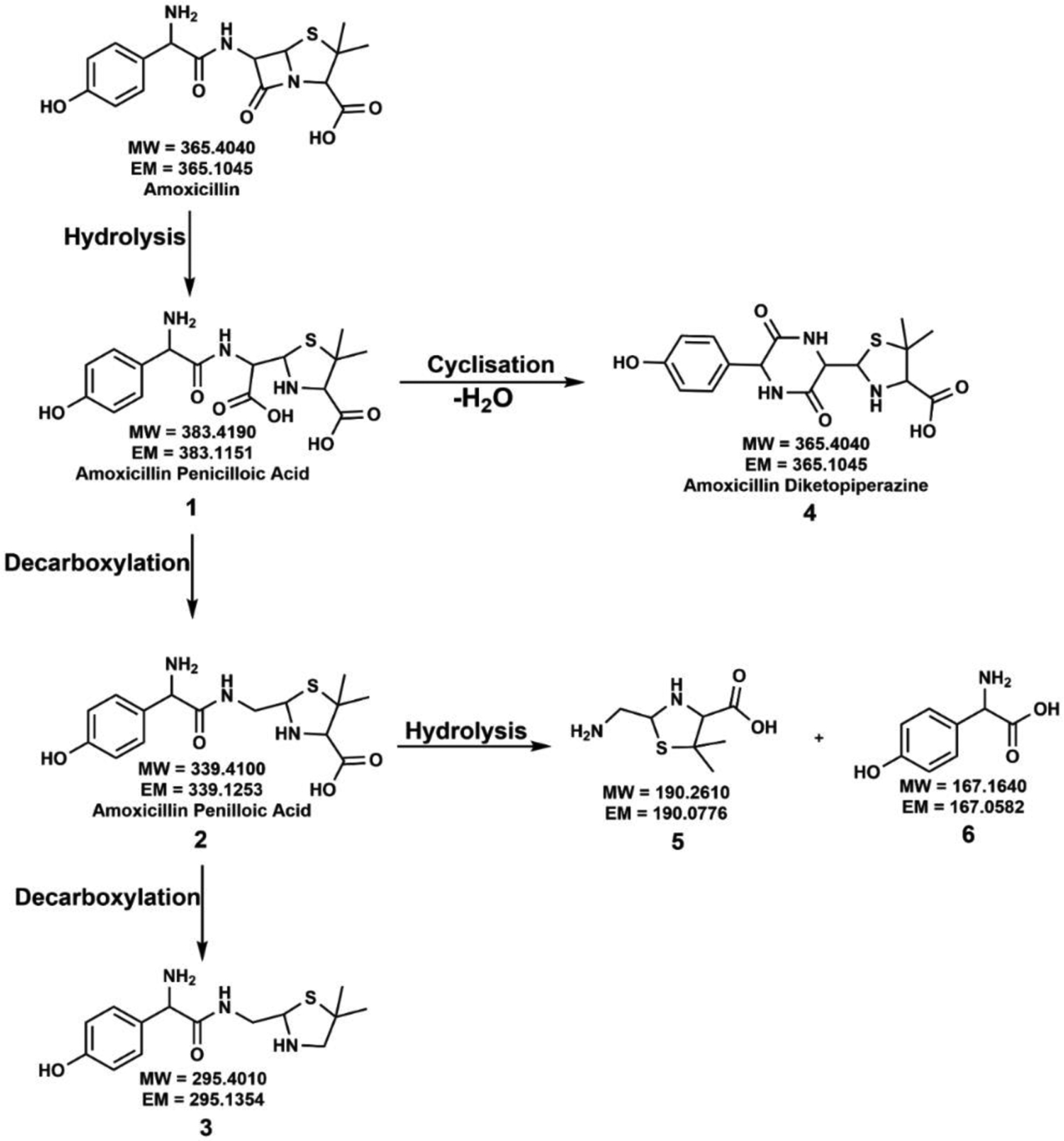
Proposed degradation mechanism of amoxicillin in the absence of mineral surface under dark and terrestrial solar radiation (AM 1.5G filter)

The proposed degradation pathways of amoxicillin degradation under dark and light conditions are discussed below. Our results highlighted that the formation of degradation products is highly mineralogy-controlled. Here, we will compare all the proposed degradation mechanisms with the Light Control. The complete degradation pathway of amoxicillin with the proposed degradation products in the absence of a mineral surface is summarized in Figure 2.

The degradation of amoxicillin in an acidic pH starts with the opening of the strained four-membered beta-lactam ring to yield the hydrolysis by-product **1**, amoxicillin penicilloic acid [14,16,34,41,45–49]. Compound **1** can undergo two possible degradation pathways to yield several different transformed compounds. In one path, the decarboxylation of the free carboxylic acid of compound **1** leads to the formation of stereoisomeric compound **2**, amoxicillin penilloic acid [14,16,34,41,46–48]. The subsequent release of the carboxylic group from the penilloic acid generates the degradation compound **3**. Another possible degradation pathway of compound **1** is forming a new, stable, six-membered ring, amoxicillin diketopiperazine, compound **4** via cyclization reaction after removing a water molecule [14,16,34,45,49,50]. Compound **2**, amoxicillin penilloic acid, can undergo hydrolysis at the amide bond, leading to the formation of another two amoxicillin degradation products, **5** [49] and **6**. Several of the above degradation products have been previously reported for actual environmental samples. For example, amoxicillin penicilloic acid was detected in Israel’s secondary effluents, and amoxicillin diketopiperazine was detected in groundwater in Israel [14].

After introducing kaolinite into the reaction mixture, we observed the formation of a fewer number of degradation products compared to the Light Control. According to Figure S3, irradiation of amoxicillin in the presence of kaolinite produced only compounds **1**, **2**, and **3**. Here, kaolinite did not facilitate the cyclization reaction of compound **1**, amoxicillin penicilloic acid, to form amoxicillin diketopiperazine, compound **4**. Further, we did not observe the hydrolysis of compound **2**, amoxicillin penilloic acid, to form compounds **5** and **6**. As explained above, light penetration is limited after introducing kaolinite compared to the Light Control. Hence, limited energy for amoxicillin molecules to undergo further degradation could be the reason for forming fewer transformed products.

**Figure 3.**
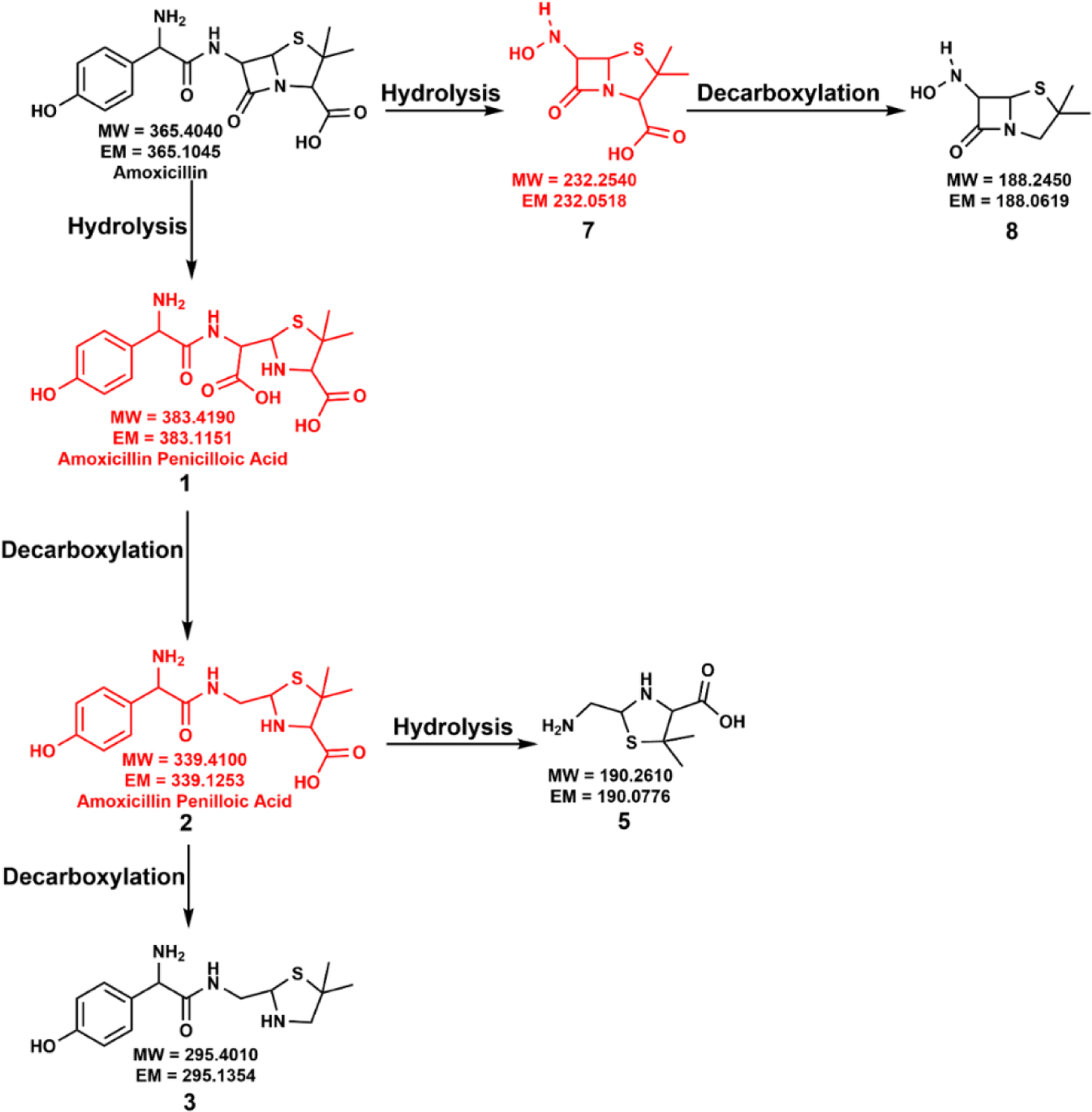
Proposed degradation mechanism of amoxicillin in the presence of anatase under irradiated conditions using AM 1.5G filter. Red-colored compounds were not detected in this work.

Interestingly, we observed significant differences in the formation of amoxicillin degradation products after introducing anatase into the reaction mixture (Figure 3). It is worth highlighting that anatase facilitated a different amoxicillin degradation pathway that was not observed in the Light Control or kaolinite reaction. Hydrolysis of amoxicillin amide bond was observed to produce intermediate **7**, and subsequent decarboxylation of intermediate **7** produced the degradation compound **8**. However, intermediate **7** was not detected in our LC-MS analysis due to the rapid degradation in the presence of the anatase surface. Apart from the amoxicillin amide bond hydrolysis pathway, amoxicillin degradation via hydrolysis of the β-lactam ring was also observed, similar to the Light Control and kaolinite reactions

Only compound **3** was observed on the 14th day, while compounds **1** and **2** disappeared. Though the hydrolysis of the amide bond of compound **2** was observed, only compound **5** was detected in the reaction mixture on the 14th day, while compound **6** disappeared. Furthermore, compound **4**, amoxicillin diketopiperazine, was not observed. The high catalytic effect of anatase leads to fast degradation of these intermediates, which could be attributed to the disappearance of some intermediates.

### 3.5. Amoxicillin Degradation under Dark Conditions

In the Dark Control, compound **1**, amoxicillin penicilloic acid, was the only degradation product observed. Similar to the Dark Control, the kaolinite reaction in the dark also showed the formation of only compound **1** as an amoxicillin degradation compound.

Interestingly, anatase showed significant differences in the formation of amoxicillin degradation products under dark conditions compared to the Dark Control and kaolinite dark reactions. The proposed reaction mechanism is illustrated in Figure S4. We observed that anatase could facilitate the formation of compounds **1**, **2**, **3**, **5**, and **6** under non-irradiated conditions. The catalytic effect of the anatase surface via surface hydroxyl groups could facilitate the formation of these compounds. However, it did not form compound **4**, amoxicillin diketopiperazine, the cyclization product of compound **1**. It should be highlighted that anatase can produce similar amoxicillin degradation compounds under dark conditions similar to the Light Control except for compound **4**. These observations further reveal the significant impact of the anatase on environmental amoxicillin degradation.

### 3.6. Amoxicillin Degradation with High-Intensity Light (AM 0 Filter)

**Figure 4.**
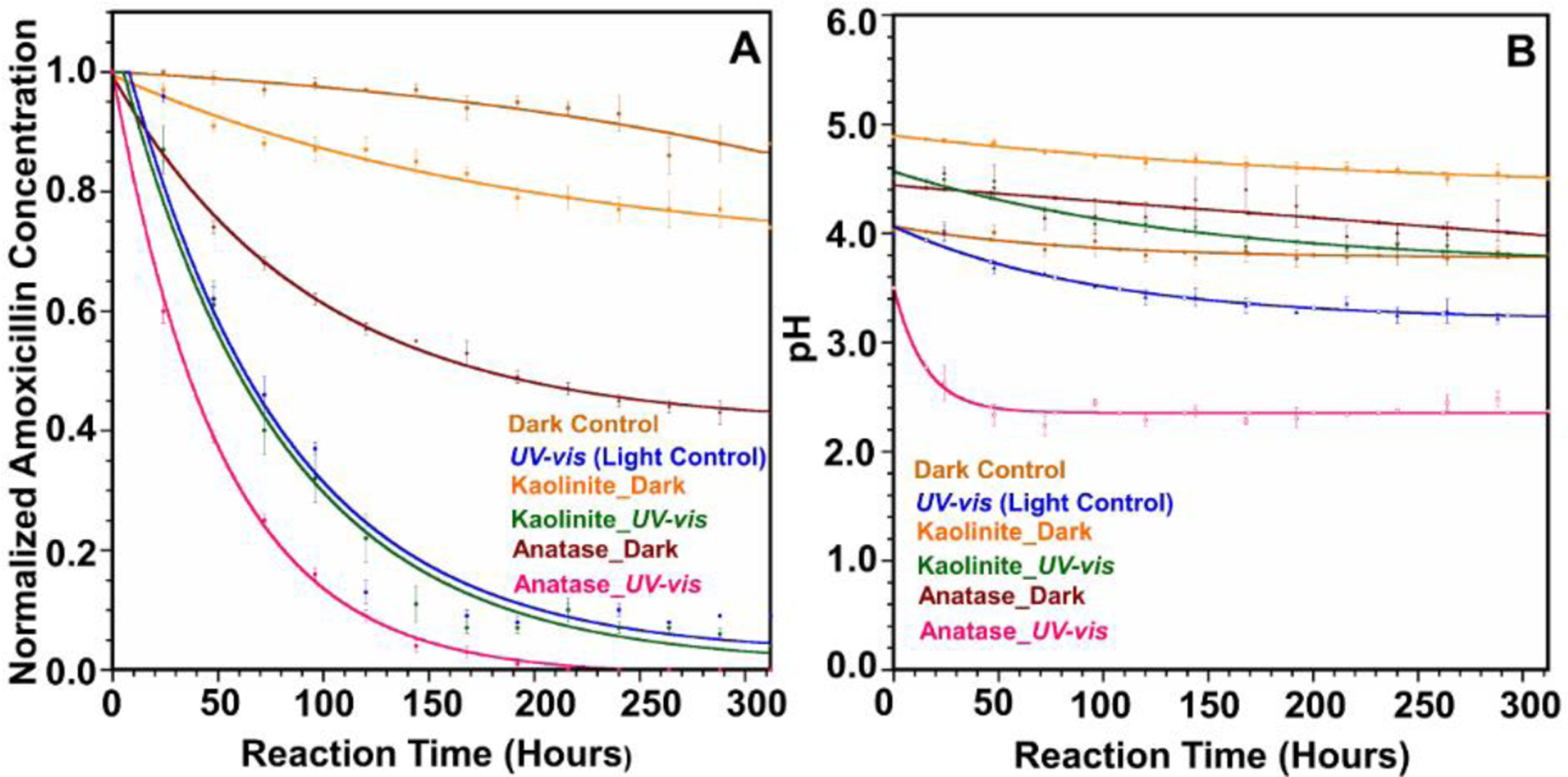
(**A**) Decay curve of amoxicillin with anatase and kaolinite under AM 0 filter light and dark conditions. (**B**) pH variation of amoxicillin experiments under AM 0 filter light. The Dark Control, Kaolinite Dark, and Anatase Dark are the same decay curves presented in Figure 1 and Table 1, and included here for comparison.

Further, we continued our experiments by irradiating amoxicillin under high-intensity light using AM 0 filter in the solar simulator. Our primary focus in these experiments was to understand the possibility of using high-intensity light to remove amoxicillin from wastewater. These decay curves and kinetics of amoxicillin degradation under these conditions are shown in Figure 4. Here, the Dark Control, Kaolinite Dark, and Anatase Dark are the same decay curves presented in Figure 1 and Table 1, and included in Figure 4 for comparison.

We observed that amoxicillin degradation rates were significantly increased in every case. Similar to the terrestrial light, anatase showed the highest degradation rate of amoxicillin (k = 0.0206 h^−1^). This is a 2.7-fold increase compared to amoxicillin degradation under terrestrial light when mixed with anatase. The rate of degradation of amoxicillin in the Light Control was 0.0140 h^−1^ which is an 8-fold increase from the terrestrial light. In the presence of kaolinite, the amoxicillin degradation rate showed a 12.8-fold increase compared to the AM 1.5G filter light. Even though the degradation rate of amoxicillin was significantly increased with high-intensity light, we did not observe mineralization of amoxicillin in any case. Interestingly, LC-MS analysis conformed formation of degradation products for high-intensity light different from terrestrial light. These degradation compounds’ ecological impacts and human health effects are not well understood yet, and thus it will limit the use of high-intensity light in removing amoxicillin from wastewater. The observed rate constants, R_2_, and half-life values for amoxicillin with high-intensity light are given in Table 2.

### 3.7. Amoxicillin Degradation Mechanisms with High-Intensity Light

**Figure 5.**
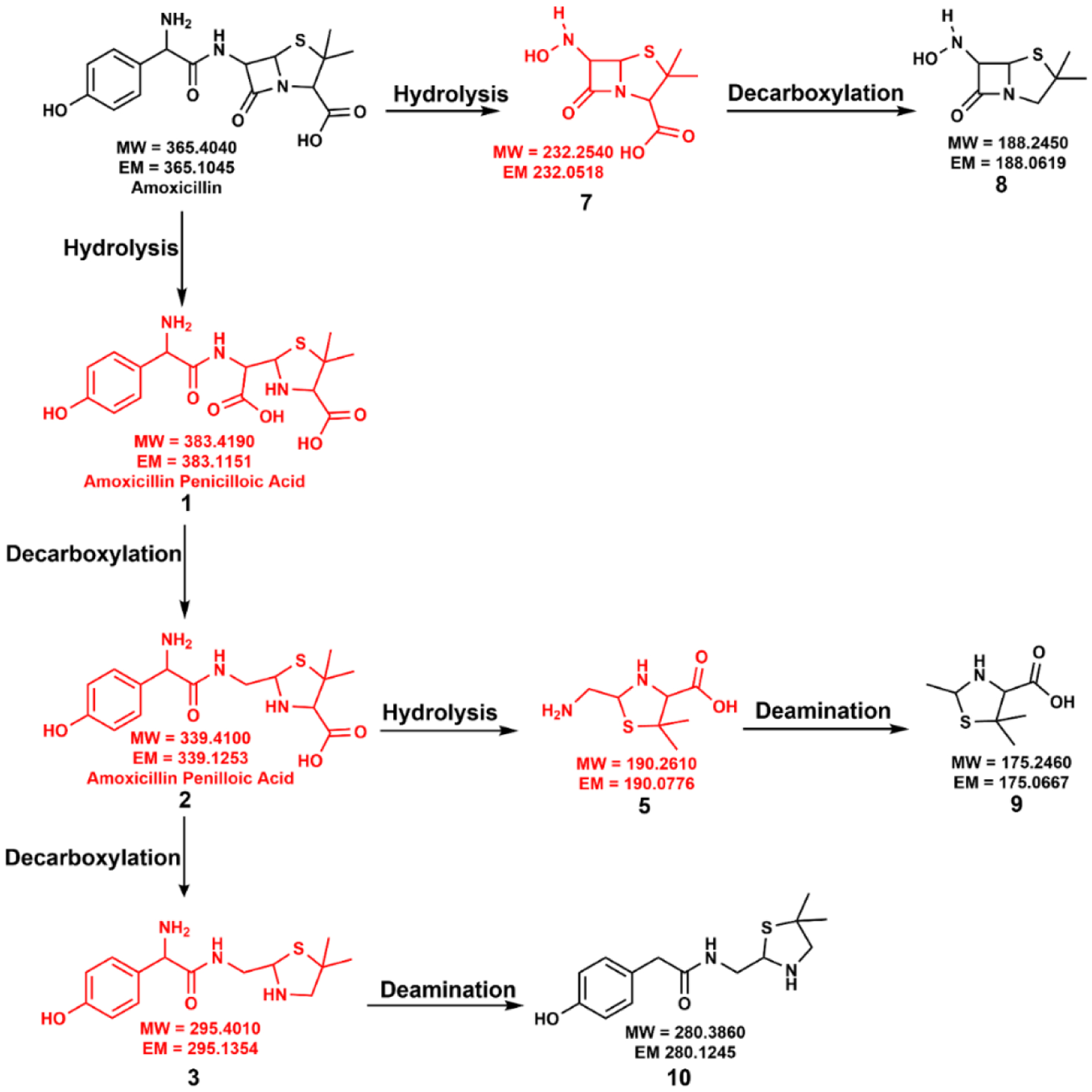
Proposed degradation mechanism of amoxicillin in the presence of anatase and high-intensity light (AM 0 filter). Red-colored compounds were not detected in this reaction.

Our results highlight the formation of several degradation products of amoxicillin under high-intensity light (Figure 5). When high-intensity light was used in the Light Control, the amoxicillin degradation rate increased significantly compared to the terrestrial light. However, we did not observe differences in the formation of degradation products with a high-intensity light. Instead, the exact degradation mechanism for amoxicillin was observed to be similar to the terrestrial solar spectrum, as depicted in Figure 2.

In contrast, irradiation of amoxicillin with high-intensity light and kaolinite showed extended degradation compared to terrestrial light. Apart from degradation compounds **1**, **2**, and **3** here, we observed the formation of compounds **4**, **5**, and **6**, which were not detected under terrestrial light (Figure S3). The amount of available photons for amoxicillin molecules to degrade is higher with the AM 0 filter than with the AM 1.5G filter, and that could be the reason for the formation of compounds **4**, **5**, and **6** under AM 0 filter.

Excitingly, in the presence of anatase, we observed significant differences in the formation of degradation compounds with a high-intensity light. The proposed degradation mechanism in the presence of anatase and high-intensity light (AM 0 filter) is provided in Figure 5. Amoxicillin was able to undergo deamination reactions with AM 0 filter light which were not observed in any of the other cases. Here, we observed the formation of two new degradation compounds, **9** and **10**, via deamination reactions. Hence, compound **5** undergoes deamination to produce compound **9**. Additionally, compound **3** yields compound **10** after a deamination reaction.

Similar to the anatase reaction with terrestrial light, amoxicillin undergoes a hydrolysis reaction at the amide bond to form intermediate **7** and subsequent decarboxylation to produce degradation compound **8**. Here, amoxicillin also undergoes the usual β-lactam ring hydrolysis pathway. Due to amoxicillin’s significantly high degradation rate with anatase and high-intensity light, we did not observe compounds **1**, **2**, **3**, **5**, and **7** in the final reaction mixture. Overall, we observed amoxicillin degradation compounds **8**, **9**, and **10** in the final reaction mixture in the presence of anatase with a high-intensity light. It should be highlighted that compounds **9** and **10** formed via deamination reactions were only observed in high-intensity light and anatase.

Based on our results, we confirm that amoxicillin can degrade significantly faster with a high-intensity light. However, the use of high-intensity light to remove amoxicillin from wastewater is still a concern due to the formation of considerable degradation products in high-intensity light and minerals. Besides, the toxicological impacts of the observed amoxicillin degradation products are not well understood yet and are strongly recommended for future studies.

## 4. Conclusions

Our study investigated the photolytic degradation of β-lactam antibiotic amoxicillin upon exposure to light and ecological minerals anatase and kaolinite. Here, we proposed a combination of direct photolysis and indirect photodegradation mechanisms for amoxicillin in the environment. Our study highlighted for the first time that the rate of amoxicillin degradation could be varied with the exposed mineral surface, while anatase caused the highest degradation rate. On the other hand, solar flux under ecological conditions was not sufficient to activate the kaolinite as photocatalyst. Thus, the material acted only as an adsorbent. Moreover, the formation of degradation compounds also varied with the available mineral surface. Light Control formed the compounds **1**, **2**, **3**, **4**, **5**, and **6**, and kaolinite formed only compounds **1**, **2**, and **3**. Anatase formed compounds **3**, **5**, and **8**.

In dark reactions, the effect of anatase was significant on amoxicillin degradation. However, the Light Control and kaolinite mineral presence caused a slow degradation. In terms of amoxicillin degradation compounds, anatase formed compounds **1**, **2**, **3**, **5**, and **6,** while Light Control and kaolinite reactions produced only compound **1**. It reveals that environmental mineralogy can play a significant role in the degradation of amoxicillin and the formation of degradation products regardless of the light availability.

Further, we irradiated amoxicillin with a high-intensity light to understand the possible method to remove amoxicillin from wastewater. Here, we observed that amoxicillin degraded significantly faster with high-intensity light than in terrestrial light—however, the formation of different amoxicillin degradation products limits high-intensity light to remove amoxicillin from wastewater. Here, anatase formed compounds **8**, **9**, and **10**. Light Control and kaolinite reactions formed **1**, **2**, **3**, **4**, **5**, and **6**. The toxicological impacts of these degradation compounds can be very different from the parent pharmaceutical amoxicillin on human health and environmental species. In further research, we suggest investigating the effects of amoxicillin degradation products in the short term and long term.

## Supplementary Material

Supporting Information

## Figures and Tables

**Table 1.** Rate constants, the half-life of amoxicillin with different mineral surfaces, and the correlation coefficient of reactions with AM 1.5G filter.

Surface	Rate Constant (h^−1^)	R^2^	H alf-Life (h)
Light Control	1.74 × 10^−3^	0.994	398
Kaolinite Solar	1.05 × 10^−3^	0.997	660
Anatase Solar	7.73 × 10^−3^	0.992	89.6
Dark Control	0.250 × 10^−3^	0.964	2770
Kaolinite Dark	1.28 × 10^−3^	0.971	541
Anatase Dark	4.69 × 10^−3^	0.987	148

**Table 2.** Rate constants, the half-life of amoxicillin with different mineral surfaces, and the correlation coefficient of reactions with AM 0 filter.

Surface	Rate Constant h^−1^	R^2^	Half-Life (h)
Light Control	1.40 × 10^−2^	0.957	49.5
Kaolinite Solar	1.34 × 10^−2^	0.980	51.7
Anatase Solar	2.06 × 10^−2^	0.996	33.6

## Data Availability

The data presented in this study are openly available in https://1drv.ms/x/s!Am5LMLzj6fUWglAYva7SGka2UtL4 (accessed on 25 May 2022); https://1drv.ms/x/s!Am5LMLzj6fUWglJfipmewkuSzuAh (accessed on 25 May 2022).
