# Supplementary material for: Heterogeneous Photocatalysis of Amoxicillin under Natural Conditions and High-Intensity Light: Fate, Transformation, and Mineralogical Impacts
Source: Environments. Author manuscript; Available in PMC 2022 Sep 22. (PMC9498904; doi:10.3390/environments9070077)
Supplement: Supporting Information [file NIHMS1836662-supplement-Supporting_Information.pdf]

Supporting Information

Heterogeneous Photocatalysis of Amoxicillin under Natural Conditions and High-Intensity Light: Fate, Transformation, and Mineralogical Impacts

Nishanthi Ellepola and Gayan Rubasinghege*

Department of Chemistry, New Mexico Institute of Mining and Technology, Socorro, NM 87801, USA; nishanthi.ellepola@student.nmt.edu
* Correspondence: gayan.rubasinghege@nmt.edu

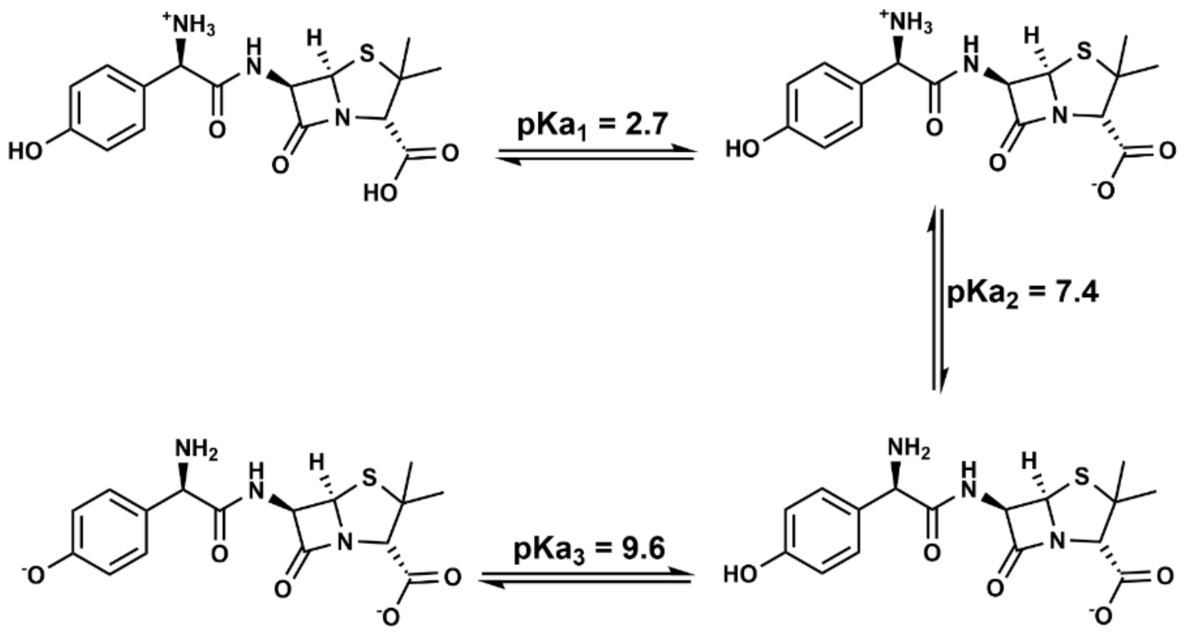

Figure S1. Different forms of amoxicillin with pKa values.

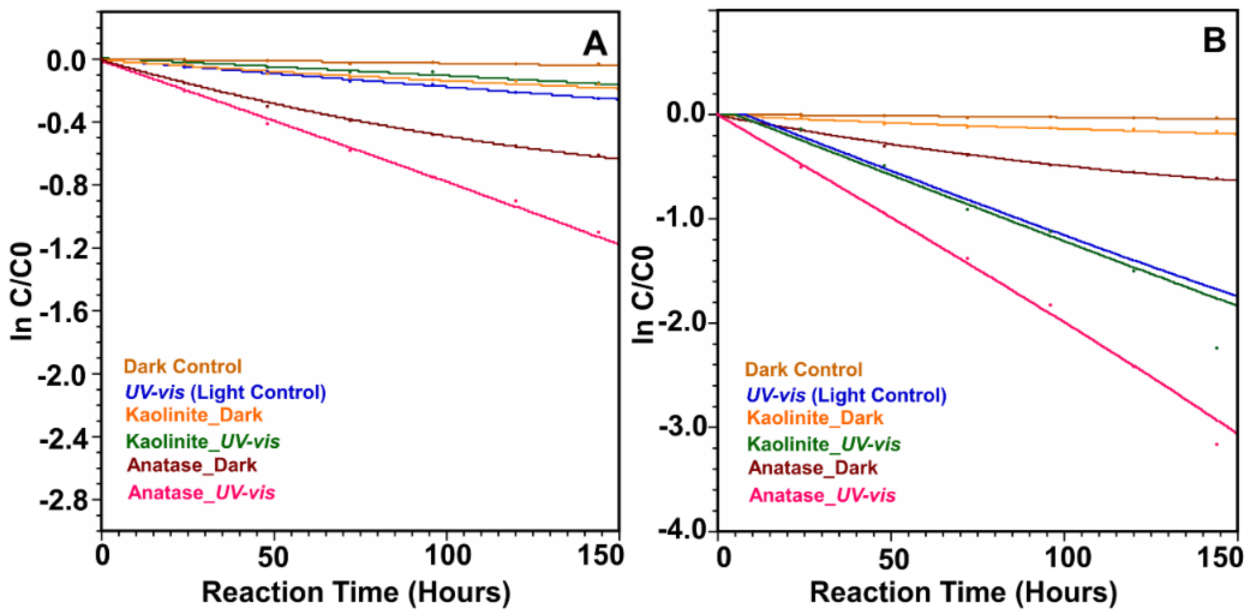

Figure S2. Kinetics of amoxicillin decay. (A) AM 1.5G filter. (B) AM 0 filter.

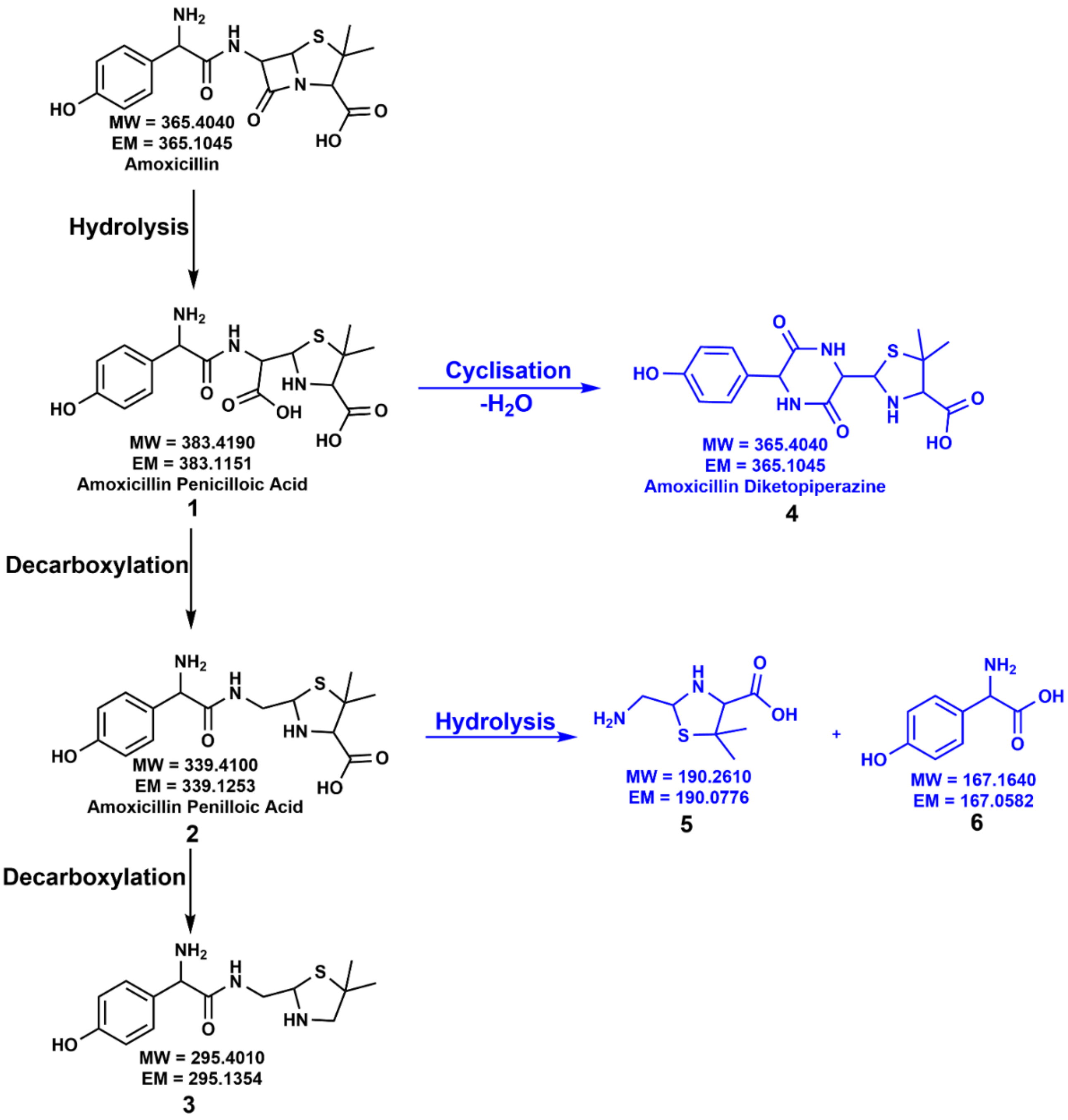

Figure S3. Proposed degradation mechanism of amoxicillin in the presence of kaolinite under irradiated conditions. Blue-colored compounds were not detected with AM 1.5G filter light.

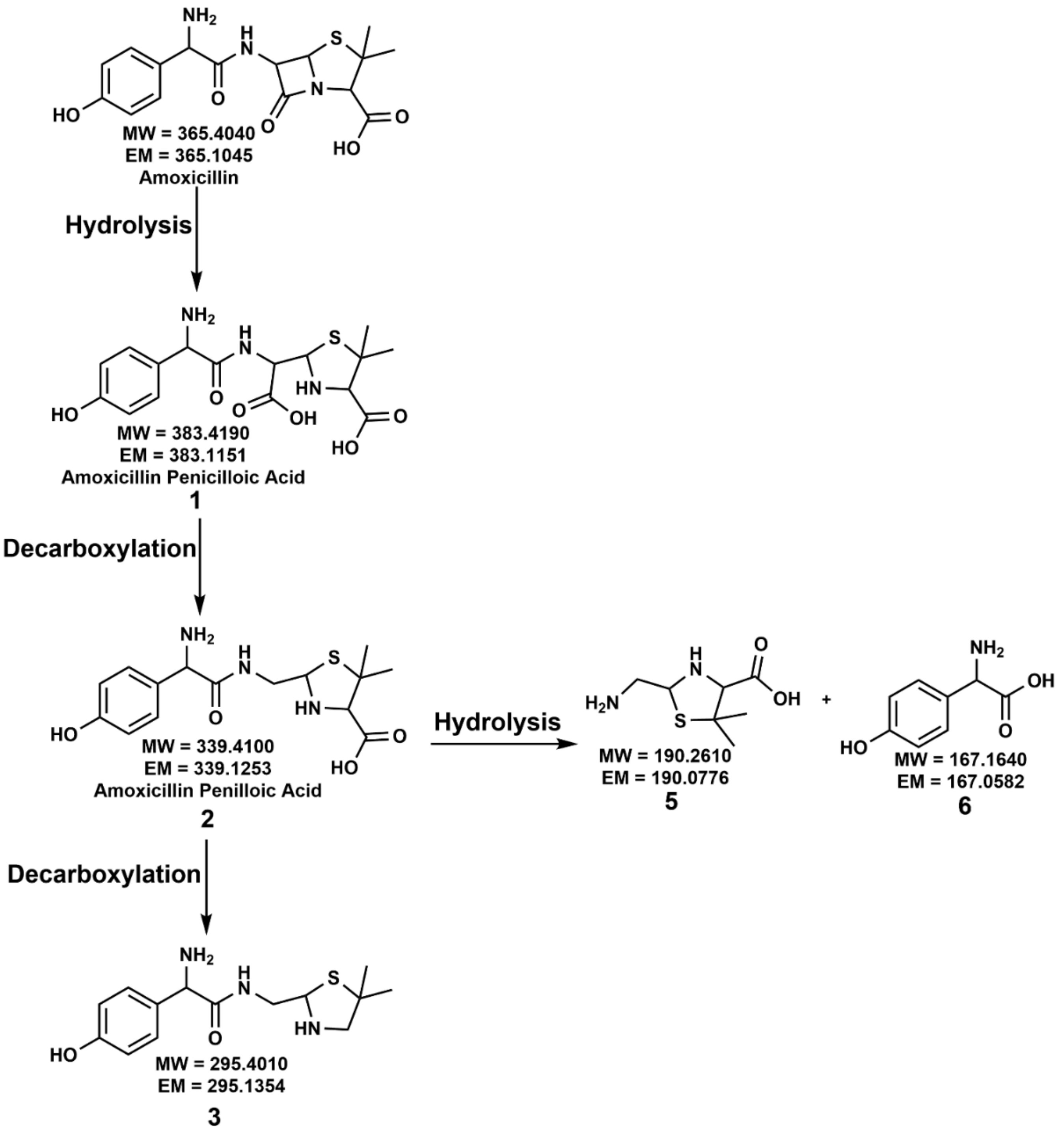

Figure S4. The proposed degradation mechanism of amoxicillin in the presence of anatase under dark conditions.

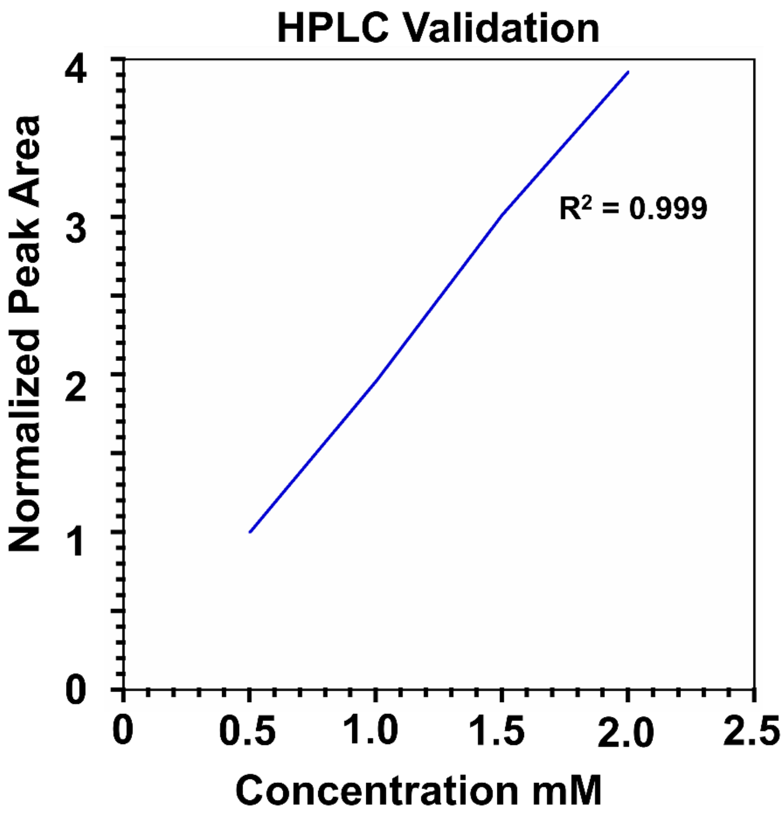

Figure S5. HPLC instrument and Reverse phase C18 column validation for linearity.

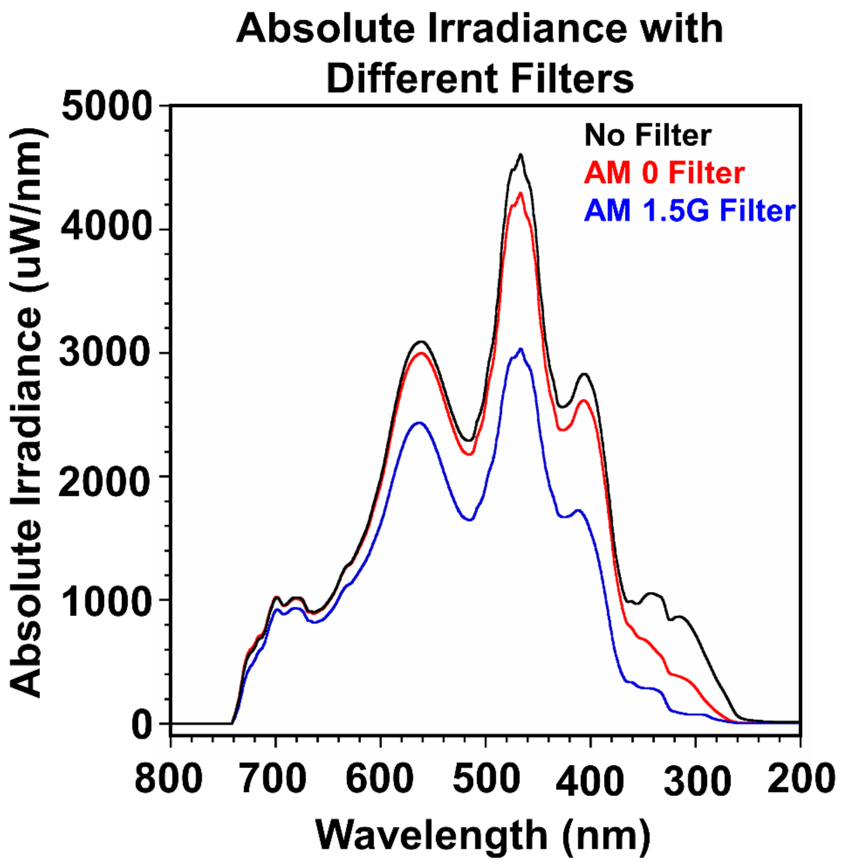

Figure S6. Solar spectrum for different filters used in the experiments.
